# Exploring gender differences in tobacco cue-induced craving and heart rate variability in individuals with a tobacco use disorder

**DOI:** 10.1016/j.dadr.2025.100407

**Published:** 2025-12-29

**Authors:** Lucia Hoffmann, Annel, P. Koomen, Taco J. De Vries, Anne Marije Kaag

**Affiliations:** a Departement of Clinical, Neuro and Developmental Psychology, Vrije Universiteit Amsterdam, the Netherlands; b Alzheimer Center Amsterdam, Department of Neurology, Amsterdam UMC, the Netherlands; c Amsterdam Neuroscience, Vrije Universiteit, Amsterdam, the Netherlands; d Department of Anatomy & Neurosciences, Amsterdam Neuroscience, Amsterdam University Medical Center, Amsterdam 1081 Hz, the Netherlands; e Institute of Brain and Behavior, Vrije Universiteit Amsterdam, the Netherlands; f Amsterdam Institute of Addiction Research, Arkin, Amsterdam, the Netherlands

**Keywords:** Gender, Cue reactivity, Heart rate variability, Craving, Reward, Relief, Nicotine

## Abstract

**Introduction:**

Women face greater challenges quitting smoking and higher health risks than men, yet gender remains understudied in tobacco use disorder (TUD). This study investigates gender differences in subjective craving and heart rate variability (HRV) following tobacco cue exposure in abstinent individuals with a tobacco use disorder. Unlike heart rate, HRV reflects parasympathetic modulation, critical for understanding risk and resilience in addiction, but has rarely been studied as a cue-reactivity biomarker.

**Methods:**

Data from 41 men and 40 women who smoked cigarettes for more than 10 years were analyzed. Participants underwent a cue-exposure paradigm consisting of a relaxation phase (75 s), exposure to smoking videos (150 s) and pictures (150 s), and handling tobacco paraphernalia (120 s). Relief craving (the urge to use nicotine to alleviate negative emotions) and reward craving (the urge to use nicotine for its pleasurable effects) were measured pre/post cue exposure via the brief Questionnaire on Smoking Urges. HRV was continuously measured as root mean square of successive differences (RMSSD).

**Results:**

Cue exposure increased relief and reward craving and reduced HRV across participants (p < .001) without gender differences. Significant craving-HRV associations emerged only in women: those with higher exposure-induced reward craving showed the largest HRV reductions and recovery during paraphernalia handling (p < 0.01), whereas those with higher exposure-induced relief craving had smaller HRV declines and weaker recovery (p < 0.01).

**Conclusion:**

These findings reinforce HRV as a clinically relevant biomarker for tobacco cue reactivity and highlight gender differences in the autonomic nervous system’s role in craving among individuals with TUD, suggesting stronger involvement in women.

## Highlights

• Tobacco cues increased reward/relief craving and reduced HRV in abstinent individuals with a tobaccu use disorder.
• No gender differences appeared in subjective or physiological cue reactivity.
• Links between cue-induced craving and HRV were observed in women, but not in men.
• Women with highest cue-induced reward craving had largest cue-induced HRV reductions.
• Women with lowest cue-induced relief craving had largest cue-induced HRV reductions.

## 1 Introduction

Tobacco use disorder (TUD) is defined as a problematic pattern of tobacco use leading to significant impairment or distress and is characterized by symptoms including loss of control, craving, withdrawal, and continued use despite negative consequences (American Psychiatric Association, 2013, Hasin et al., 2013, Shmulewitz et al., 2022). Smoking remains the leading cause of preventable illness worldwide (Tobacco EURO, n.d.). Importantly, the burden of smoking appears greater in women than in men: women who smoke have higher risks of lung cancer, chronic obstructive pulmonary disease, and coronary heart disease (McKee and McRae-Clark, 2022, Menson and Coleman, 2024, Tramunt et al., 2023). Despite many expressing the intention to quit, only about a quarter succeed (Smit et al., 2014). Notably, women with TUD are less responsive to nicotine replacement therapies (Verplaetse et al., 2018) and experience stronger nicotine withdrawal symptoms than men (Becker et al., 2017, Conti et al., 2020, Weinberger and Platt, 2016) contributing to poorer cessation outcomes (Castaldelli-Maia et al., 2022, Weinberger and Platt, 2016). Gaining a deeper understanding of potential distinctions between men and women in the mechanisms underlying TUD is crucial for developing gender-tailored interventions. Throughout this article, we will refer to differences between men and women as gender differences, as both biological characteristics related to sex assigned at birth as well as environmental, sociocultural and developmental factors related to gender, are likely to contribute to these differences (Becker et al., 2017, Fonseca et al., 2021).

A key characteristic of TUD is cue reactivity, which refers to the physiological and subjective reaction to drug-associated stimuli (Karelitz, 2019). Subjective tobacco cue-reactivity (craving) significantly predicts nicotine behaviors like latency to smoke and smoking quantity (Conklin et al., 2015). While some studies have found greater tobacco cue-induced craving in women than in men (Carpenter et al., 2014, Doran, 2014, Field and Duka, 2004), most find no gender difference (Betts et al., 2021, Colamussi et al., 2007, McClure et al., 2020, Pericot-Valverde et al., 2015, Saladin et al., 2012, Wray et al., 2015). Inconsistencies may arise because most studies measure craving with single-item questions such as “How much do you crave a cigarette right now?”, which emphasize reward-related craving (i.e. the urge to use nicotine for its pleasurable effects) (Colamussi et al., 2007, McClure et al., 2020, Pericot-Valverde et al., 2015, Wray et al., 2015, Zanchi et al., 2016), while craving can also be related to relief (i.e., the urge to use nicotine to reduce negative emotions and withdrawal symptoms) (Cox et al., 2001, Dondé et al., 2020). Indeed, research in other substance use disorders (SUDs) suggests that reward craving predominates in men, while relief craving is more prominent in women (Gex et al., 2023, , & PREDICT Study Research Group, 2013). As such, it could be important to include the different aspects of craving when investigating gender differences in tobacco cue reactivity.

Beyond subjective craving, cue reactivity paradigms lead to strong physiological responses. Individuals with a SUD, including TUD, show heightened activation in the brain reward circuitry when exposed to substance-related cues, as compared to neutral cues (Kühn and Gallinat, 2011) which is linked to treatment outcomes and relapse (Courtney et al., 2016, Versace et al., 2014). Research in TUD has demonstrated gender differences in physiological tobacco cue reactivity, with studies reporting both elevated (McClernon et al., 2008, Zanchi et al., 2016) and blunted (Dumais et al., 2017, Hanlon et al., 2012) neural cue reactivity in women compared to men. Moreover, cue-induced craving is associated with neural tobacco cue reactivity in men but not in women (Dumais et al., 2017). Taken together, these neural findings highlight the complexity of gender-specific cue reactivity patterns, but they provide only part of the picture. Evidence increasingly supports the role of the autonomic nervous system (ANS) in SUDs, including TUD (LaFond et al., 2024, Milivojevic and Sinha, 2018). A key ANS biomarker is heart rate variability (HRV), which reflects the variation in time between consecutive heartbeats (Rajendra Acharya et al., 2006). Unlike heart rate (HR) HRV specifically captures dynamic parasympathetic (vagal) modulation, which is critical for understanding both risk and resilience in addiction (Garland et al., 2012, Ralevski et al., 2019, Wang et al., 2020). HRV can be measured in the frequency domain or via the time domain using the root mean square of successive differences between heartbeats (RMSSD) (Shaffer and Ginsberg, 2017). Higher RMSSD and high-frequency HRV indicate greater parasympathetic (vagal) activity, associated with relaxation. Conversely, lower RMSSD and low-frequency HRV suggest reduced vagal activity, often linked to stress (Rajendra Acharya et al., 2006). RMSSD is particularly suitable for short-term HRV measurement (Shaffer and Ginsberg, 2017) and could thus be considered a relevant biomarker of tobacco cue reactivity to identify neurobiological processes that lead to the development and symptomatology of TUD (Milivojevic and Sinha, 2018).

While various studies demonstrated that HR remains unchanged during substance cue-exposure (Carpenter et al., 2009, Cortese et al., 2015, Erblich and Bovbjerg, 2011, LaRowe et al., 2007, Tong et al., 2007, Upadhyaya et al., 2006), to date only one study investigated changes in HRV following tobacco cue exposure showing that such exposure leads to a decrease in high-frequency HRV indicating reduced vagal activity (Kroczek et al., 2024). Research in other SUDs, using RMSSD as an HRV metric, has yielded mixed findings. Some studies report increases in HRV during alcohol cue exposure in men and women (Ingjaldsson et al., 2003, Rajan et al., 1998), while others find decreases in HRV (Firdaus et al., 2025, Garland et al., 2012). Increased HRV is typically interpreted as a marker of attentional or emotional engagement, whereas decreased HRV is linked to stress (Ralevski et al., 2019). Gender differences might explain previous inconsistent findings, as exposure to substance cues appears to engage reward-related processes more strongly in men and relief- or stress-related processes more strongly in women (Colamussi et al., 2007, Dumais et al., 2017, Hanlon et al., 2012, McClernon et al., 2008, McClure et al., 2020, Saladin et al., 2012, Tomko et al., 2020, Versace et al., 2014, Zanchi et al., 2016). Consequently, it can be hypothesized that reward-driven craving and smoking behavior, associated with tobacco cue-induced increases in HRV, is more prominent in men, while relief-driven craving, linked to tobacco cue-induced decreases in HRV, is more pronounced in women. Given earlier inconsistent findings, this study aims to clarify gender differences in subjective and physiological (using RMSSD as an HRV metric) tobacco cue reactivity, as well as their association with each other. Given withdrawal symptoms influence craving and physiological cue responses (McClernon et al., 2008), exploratory analyses will also be performed to investigate whether (gender differences in) nicotine cue reactivity are moderated by withdrawal symptoms.

## 2 Methods

This study conducted a cross-sectional analysis of baseline data from a randomized controlled experimental lab study to test the effects of 3MDR treatment (Multi-modal Motion-assisted Memory Desensitization and Reconsolidation) on smoking cessation of which the results are published elsewhere (Koomen et al., 2024). For this intervention to be effective, we required subjects to be abstinent for 48 h as this is suggested to induce stronger craving, withdrawal and salient smoking-related memories crucial for making these maladaptive memories susceptible to reconsolidation interventions, including 3MDR (Germeroth et al., 2017). For the main study, participants had to come to the research center on five consecutive days, each of which lasted approximately 60 min. During session 1 and 5 tobacco cue reactivity was assessed; during session 2–4 participants underwent the 3MDR intervention. The current study only analyzed data from the first session.

### 2.1 Participants

Individuals who smoke cigarettes chronically (≥10 cigarettes per day for at least 10 years; age 25–55) were recruited through social media and local advertisements in the Amsterdam area, The Netherlands. Participants were instructed to abstain from using nicotine-containing substances 48 h before the start of the study. Exclusion criteria included a positive smokerlyzer (carbon monoxide) breath test; neurological disorders; a lifetime diagnosis of, or treatment for, psychosis or mania; psychiatric diagnosis or treatment within the past year; current psychotropic medication use and current substance dependence other than nicotine, indicated by Alcohol Use Disorder Identification Test (AUDIT) scores > 12 (Saunders et al., 1993) or Drug Use Disorder Identification Test (DUDIT) scores > 12 (Hildebrand, 2015). All participants provided online informed consent for prior to screening and written informed consent for the full study prior to the first lab assessment. The study was approved by the Medical Ethics Review Committee (METC) of Amsterdam UMC and was preregistered in the Dutch Trial Register (https://www.onderzoekmetmensen.nl/en/trial/55689). Participants received €20,- for each completed session (all participants completed all five sessions).

### 2.2 Procedure

After providing informed consent, individuals interested in study participation received an online screening form, assessing the inclusion and exclusion criteria. During the first in-person appointment, written informed consent was obtained from participants. Smoking abstinence was measured at the start of the session by measuring exhaled carbon monoxide levels with a calibrated smokerlyzer (Micro+) breath test, and non-abstinent (>10 ppm) participants were excluded from further study participation. Participants who tested negative for recent smoking then completed the questionnaire. Afterward, these participants were prepared to undergo the tobacco cue exposure task as planned.

### 2.3 Tobacco cue exposure task

A modified smoking cue exposure paradigm based on Kaag et al. (2018) was used to elicit tobacco craving. The task was divided into four parts. First, participants underwent a 75-second audio-guided relaxation before cue exposure. Next, they viewed five 30-second video clips of individuals of both sexes engaging in smoking-related behaviors (e.g., lighting up a cigarette, and smoking), followed by the presentation of 20 random tobacco-related images of cigarettes, individuals smoking cigarettes, and cigarette packs, each displayed for 7.5 s. The entire visual cue exposure section lasted 5 min, with variation in the clips and images presented to participants. Both the videos (Germeroth et al., 2017) and images (Kuhns et al., 2021) have been validated and used elsewhere.

After the visual cues, participants received computerized instructions to handle tobacco-related items, including a cigarette pack and a lighter. They were instructed to open the pack, remove a cigarette, hold it, bring it to their face, and smell it. Finally, participants were asked to turn on the lighter as if about to light the cigarette but without actually doing so. This handling phase lasted 2 min. Subjective craving for tobacco was measured both directly pre tobacco cue exposure (following the guided-relaxation) and directly post the tobacco cue exposure using the 10-item brief Questionnaire on Smoking Urges (QSU-brief) (Cox et al., 2001).

### 2.4 Measures

Inclusion and exclusion criteria were assessed using the Dutch versions of the AUDIT and DUDIT via an online screening (Saunders et al., 1993). Both sex assigned at birth (male, female, other) and current gender identity (man, woman, non-binary) were assessed. In all cases, sex assigned at birth aligned with the individuals gender-identity. The State-Trait Anxiety Inventory (STAI) measured affective symptomatology and anxiety (Spielberger, 1983). Nicotine dependence severity over the past year was measured with the Fagerström Test for Nicotine Dependence (FTND) (Heatherton et al., 1991, Meneses-Gaya et al., 2009). Daily cigarette consumption was calculated using the nicotine Timeline Follow-Back method, based on self-reported cigarette use over the previous 14 days (Sobell and Sobell, 1992). Motivation to quit smoking was assessed using the Readiness to Change Questionnaire (Rollnick et al., 1992). Nicotine withdrawal symptoms were measured using the Minnesota Nicotine Withdrawal Scale (MNWS) (Toll et al., 2007). For hormonal contraceptive users, the contraceptive type was recorded; for naturally cycling women, average cycle length and days since last menstruation were documented.

To assess tobacco cue-induced craving, the Dutch translation of the brief Questionnaire of Smoking Urges (QSU) was used (Cox et al., 2001) which distinguishes between craving related to relief (items 2, 4, 5, 8, 9) and reward (items 1, 3, 6, 7, 10). Craving was assessed directly prior to the tobacco cue exposure paradigm and directly following the tobacco cue exposure paradigm.

HRV, as the physiological measure of cue reactivity, was measured during four different conditions: relaxation, exposure to video, picture tobacco cues, and handling of physical tobacco-related cues. HRV was recorded with the Vrije Universiteit Ambulatory Monitoring Solution (VU-AMS) device version 5.4.13 and processed and visualized with the Vrije Universiteit – Data Analysis and Management (VU-DAMS) program version 5.4.13 (VU-DAMS, n.d.).

### 2.5 Data Analysis

#### 2.5.1 Demographic and clinical characteristics

One-way ANOVAs were used to test for differences between men and women in age, daily cigarette use, tobacco use severity (using the FTND), alcohol use severity (using the AUDIT), drug use severity (using the DUDIT), state anxiety (using the state STAI), and nicotine withdrawal symptoms (using the MNWS). Chi-square tests assessed gender differences in drug use over the past year (using the DUDIT) and readiness to change smoking behavior. Significant gender differences in these variables were examined in follow-up analyses to assess whether they confounded subjective or physiological cue reactivity results.

#### 2.5.2 Preprocessing of physiological data

ECG data were preprocessed using VU-AMS software (https://vu-ams.nl), which automatically detects R-peaks and removes large ECG artifacts. Smaller deviations are flagged for manual inspection and correction if necessary. The time intervals between successive R-peaks (inter-beat intervals, IBI) are then calculated to generate the IBI time series. RMSSD is computed as the root mean square of successive differences between adjacent R-R intervals: the differences in milliseconds are squared to make them positive, averaged, and then the square root is taken. This semi-automated procedure combines robust automatic detection with visual quality control to ensure high-quality heart rate data.

#### 2.5.3 Interferential analyses

Repeated measures ANOVAs in SPSS assessed the effects of tobacco cues on self-rated relief and reward cravings, and HRV. Assumptions, including sphericity and normality, were tested using Mauchly’s and normality tests; slight deviations from normality were accepted given the adequate sample size.

Relief and reward craving scores pre- and post-cue exposure served as within-subject variables for craving analyses. HRV within-subject variables included relaxation, cue video, cue picture, and cue handling. The interaction between cue-induced HRV changes and craving changes was examined via repeated measures ANOVA, entering craving difference scores (post minus pre) as covariates.

In all analyses, gender was included as a between-subjects factor to examine gender effects. The best-fitting model (linear, quadratic, or cubic) was reported, with follow-up analyses for significant interactions. Exploratory analyses tested whether gender differences in subjective and physiological cue reactivity were influenced by self-reported withdrawal symptoms. Because of the exploratory nature of the study, no correct for multiple comparisons was applied.

## 3 Results

### 3.1 Descriptive analyses

Men and women in the sample were similar in age and reported comparable daily cigarette use, tobacco use severity, nicotine withdrawal, alcohol severity, state anxiety, and readiness to change. Men reported significantly higher drug use in the past 12 months than women. Among those reporting drug use, severity scores were low and similar between men and women. Among all women, 41 % used hormonal contraceptives, mostly hormonal IUDs. Half of non-contraceptive-using women reported regular menstrual cycles. See Table 1 for detailed demographic and statistical information. Exploratory analyses on the influence of hormonal contraceptive use on tobacco cue reactivity are described in the Supplementary Materials.

**Table 1:** Nicotine use characteristics of men and women with nicotine use disorder.

	Men (n = 40)	Women (n = 41)	Gender-differences
Age	41.25 ± 8.10	42.15 ± 8.18	F= 0.245, p = 0.622, η^2^ < 0.01
Number of daily cigarettes^1^	15.38 ± 5.93	13.74 ± 5.93	F_1,81_= 1.55, p = 0.22, η^2^= 0.02
Tobacco Use Severity (FTND)	4.98 ± 2.11	4.29 ± 2.15	F_1,81_= 2.08, p = 0.153, η^2^ = 0.03
Alcohol Use Severity (AUDIT)	7.17 ± 3.43	5.49 ± 4.20	F_1,81_= 3.91, p = 0.05, η^2^ = 0.05
Nicotine withdrawal (MNWS)	15.62 ± 5.63	17.78 ± 6.32	F_1,81_= 2.64, p = 0.108,
CO ppm	3.43 ± 2.19	2.39 ± 1.32	F_1,81_= 6.65, p = 0.012
Drug use in past 12 months^2^			**Χ**^**2**^**= 6.59, p = 0.01**
No	61 % (n = 24)	85 % (n = 35)	
Yes	39 % (n = 16)	15 % (n = 6)	
Drug Use Severity^3^	4.50 ± 2.00	4.33 ± 3.01	F_1,20_= 0.02, p = 0.88, η^2^ < 0.01
State Anxiety (STAI-S)	42.13 ± 8.93	44.00 ± 9.45	F_1,81_= 0.80, p = 0.374, η^2^ = 0.01
Readiness to change			Χ^2^= 3.20, p = 0.07
Precontemplation phase	0 % (n = 0)	0 % (n = 0)	
Contemplation phase	25 % (n = 10)	44 % (n = 18)	
Action Phase	75 % (n = 30)	56 % (n = 23)	
Hormonal contraceptive use		41 % (n = 17)	
Hormonal IUD		53 % (n = 9)	
Oral contraceptive		23 % (n = 4)	
Mini-pill (progesterone only)		6 % (n = 1)	
Contraceptive injection		6 % (n = 1)	
Contraceptive implant		6 % (n = 1)	
contraceptive vaginal ring		6 % (n = 1)	
No hormonal contraceptive use		59 % (n = 24)	
No menstruation (anymore)^4^		37,5 % (n = 9)	
Varying menstrual cycle length^4^		12,5 % (n = 3)	
Menstrual cycle < 20 days^4^		8.33 % (n = 2)	
Regular menstrual cycle^4^		50 %% (n = 12)	
Average menstrual cycle length		25.42 ± 3.09	

Values shown present means ± standard deviations or frequencies and percentages.

^1^ Based on Time Line Follow Back in 14 days prior to study, except the two days directly preceding the study as participants were instructed to remain abstinent

^2^ Based on the first item of the DUDIT:

^3^ for n = 24 participants (6 women and 16 men) who indicated to have used drugs in the past 12 months)

^4^ percentage of women who do not use hormonal contraceptives

FTND: Fagerström Test for Nicotine Dependence

AUDIT: Alcohol Use Disorders Identification Test

STAI-S: State and Trait Anxiety Questionnaire - State

### 3.2 Gender differences in subjective and physiological tobacco cue reactivity

**Fig. 1:**
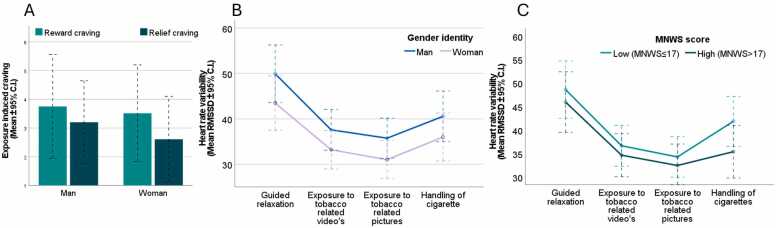
**Gender differences in tobacco cue-induced relief and reward craving, HRV and its association** While reward and relief craving significantly increased following tobacco cue exposure (A) and HRV significantly decreased during the tobacco cue exposure paradigm (B), no gender differences were apparent. (C) Withdrawal symptoms were significantly associated with tobacco-cue induced changes in HRV, with those that reported more severe withdrawal symptoms, showed a blunted increase of HRV during the handling of cigarettes. RMSSD: Root Mean Square of Successive Differences.

Both reward and relief craving significantly increased from pre-exposure to post-exposure with no moderation by gender (Fig. 1A). A significant quadratic effect of tobacco cues on HRV was observed - HRV decreased from the relaxation during the tobacco video and picture exposure, then slightly increased during handling of tobacco paraphernalia (Fig. 1B). Gender did not moderate the main effects of cue reactivity on relief craving, reward craving, or HRV. Controlling for gender differences in drug use did not change the non-significant gender differences in subjective and physiological tobacco cue reactivity. Detailed statistics are reported in Table 2.

**Table 2:** Main and interaction effects exposure and gender on heart rate variability, relief craving and reward craving.

**Heart rate variability**^**⁎**^	
Main effect gender	F_1,70_= 2.41, p = .125, η^2^= 0.03
Main effect exposure phase	**F**_**1,70**_**= 56.02, p < .001, η**^**2**^**= 0.45**^**a**^
Interaction effect exposure⁎gender	F_1,70_= 0.32, p = 0.57, η^2^< .01^b^
**Relief craving**	
Main effect gender	F_1,79_ < 0.01, p = .952, η^2^ < 0.01
Main effect exposure phase	**F**_**1,80**_**= 32.18, p < .001, η**^**2**^**= 0.29**
Interaction effect exposure⁎gender	F_1,79_ = 0.33, p = .567, η^2^< 0.01
**Reward craving**	
Main effect gender	F_1,79_ = 0.28, p = .596, η^2^ < 0.01
Main effect exposure phase	**F**_**1,80**_**= 35.77, p < .001, η**^**2**^**= 0.31**
Interaction effect exposure⁎gender	F_1,79_= 0.04, p = .846, η^2^< 0.01

^⁎^ Of all participants, 9 had missing HRV data (either due to technical failure or poor data quality) in on or more of the exposure phases. Hence, these were excluded from the HRV analysis.

^a^ Effects were best described by a quadratic trend

^b^ Effects were best described by a linear trend

Planned exploratory analyses on withdrawal symptoms demonstrated that relief (F_1,78_ =49.49, p < 0.01, η^2^= 0.39) and reward craving (F_1,78_ =13.59, p < 0.01, η^2^= 0.15) were significantly higher in individuals with more severe nicotine withdrawal, irrespective of exposure phase. However, nicotine withdrawal symptoms did not moderate the effect of tobacco cue exposure on craving. In contrast, exposure-induced HRV changes were significantly moderated by withdrawal symptoms (F_1,69_=5.24, p = 0.03, η^2^=0.07). Simple effects analyses based on a median split of MNWS scores (>17 vs. ≤17) showed that HRV changes in low-withdrawal individuals followed a quadratic trend (F_1,35_=37.18, p < 0.01, η^2^=0.52), whereas high-withdrawal individuals exhibited a linear trend (F_1,35_=32.87, p < 0.001, η^2^=0.50) (Fig. 1C).

### 3.3 Gender differences in the association between subjective and physiological tobacco cue reactivity

**Fig. 2:**
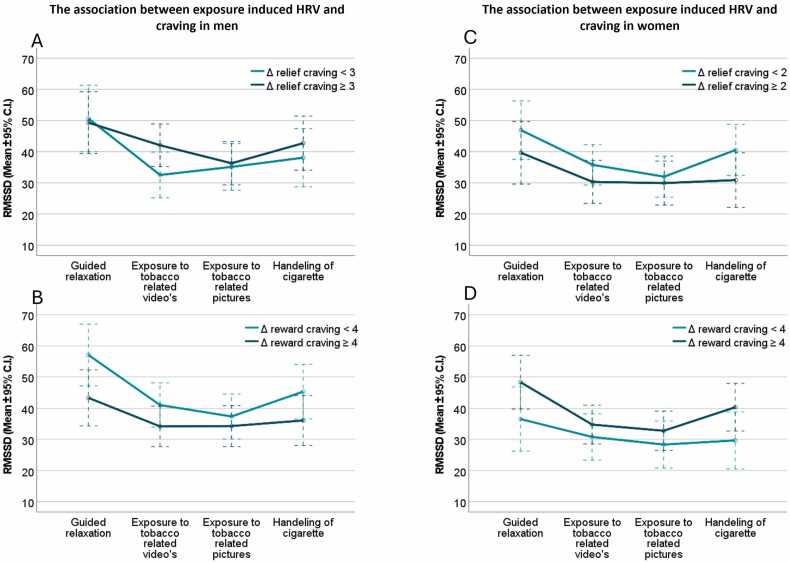
Gender differences in the relationship between tobacco cue-induced craving and changes in HRV In men, both tobacco-cue induced relief craving (A) as tobacco cue-induced reward craving (B) were unrelated to tobacco-cue induced changes in HRV. In women, tobacco cue-induced changes in HRV were significantly associated with changes in tobacco cue-induce relief craving (C) as well as tobacco cue-induced reward craving. Note: The statistical analyses used craving as a continuous variable, while the figures are based on median splits, simplifying the data. As such these figures are strictly for visualization and do not provide inferential evidence. Δcraving: change in scores on questionnaire for smoking urges, from pre to post tobacco cue exposure. RMSSD: Root Mean Square of Successive Differences.

The association between cue-induced HRV changes and both reward and relief craving was moderated by gender (Table 3; Fig. 2). Follow-up analyses showed no significant associations in men between HRV changes and craving (Fig. 2A-B). In contrast, among women, quadratic exposure-induced HRV changes were significantly related to both exposure-induced relief craving (F₁,₃₅=8.6, p < 0.01, η²=0.20) and reward craving (F₁,₃₅=9.71, p < 0.01, η²=0.22). To further characterize this interaction, simple effects analyses were conducted for women reporting low versus high exposure-induced craving, based on a median split of cue-induced craving scores. For relief craving (Fig. 2 C), a significant quadratic change in HRV was observed in women with low exposure-induced relief craving (ΔQSU relief <1; F₁,₁₈=15.21, p < 0.01, η²=0.46), while in women with high relief craving, the change was not significant and best described by a cubic trend (ΔQSU relief ≥1; F₁,₁₆=4.37, p = 0.05, η²=0.22). Significant quadratic HRV changes were observed in women with both low (ΔQSU reward<4; F₁,₁₅=27.165, p < 0.01, η²=0.64) and high (ΔQSU reward ≥4; F₁,₁₉=12.69, p < 0.01, η²=0.40) exposure-induced reward craving (Fig. 2D).

**Table 3:** Gender differences in the association between exposure-induced craving and exposure-induced changes in HRV.

**Exposure-induced relief craving**	
Main effect induced relief craving	F_1,66_ = 0.10, p = .76, η^2^< .01
Interaction effect induced relief craving ⁎ gender	F_1,66_ = 2.87, p = .10, η^2^= .04
Interaction effect exposure phase ⁎induced relief craving	F_1,66_ = 1.41, p = .24, η^2^= .02^a^
Interaction effect exposure phase ⁎ induced relief craving ⁎ gender	**F**_**1,66**_**= 4.98, p = .03, η**^**2**^**= .07**^**a**^
**Exposure-induce reward craving**	
Main effect induced reward craving	F_1,66_ = 0.28, p = .60, η^2^= <.01
Interaction effect induced reward craving ⁎ gender	F_1,66_ = 0.73, p = .40, η^2^= .01
Interaction effect exposure phase ⁎induced reward craving	F_1,66_ = 3.38, p = .07, η^2^= .049^a^
Interaction effect exposure phase ⁎ induced reward craving ⁎ gender	**F**_**1,66**_**= 4.31, p = .04, η**^**2**^**= .06**^**a**^
^a^ Effects were best described by a quadratic trend

Visual inspection suggested that women with higher cue-induced reward craving exhibited larger HRV decreases during tobacco cue exposure and greater increases during cigarette handling, compared to those with lower craving. Conversely, women with lower exposure-induced relief craving showed stronger HRV decreases during tobacco cue exposure and greater increases during handling, compared to those with higher relief craving. Planned exploratory analyses testing whether nicotine withdrawal moderated gender differences in the link between subjective and physiological tobacco cue reactivity found no significant effects. Adjusting for gender differences in drug use did not affect the primary findings regarding gender-related differences in the relationship between subjective and physiological tobacco cue reactivity.

## 4 Discussion

This study aimed to identify gender differences in subjective and physiological tobacco cue reactivity among abstinent individuals with an TUD. Exposure to tobacco cues significantly increased craving and decreased HRV, but no direct gender differences in these responses were found. However, gender differences emerged in the association between subjective craving and HRV: a significant relationship was observed only in women. Specifically, women reporting the highest exposure-induced reward craving showed the largest HRV reductions during exposure to tobacco-related images and the greatest HRV increases during cigarette handling. Conversely, women with the highest relief craving exhibited smaller HRV reductions and blunted increases during handling. These gender-related patterns are unlikely due to demographic or clinical differences, as men and women were similar in age, tobacco and alcohol use severity, nicotine withdrawal symptoms, and anxiety. Although men reported more substance use in the past year, drug use differences did not explain the gender-specific association between craving and physiological reactivity. These findings contribute to understanding how subjective and physiological cue responses interact differently by gender in individuals who smoke cigarettes during abstinence, with implications for tailored smoking cessation strategies.

In line with previous research in both abstinent and non-abstinent individuals who smoke cigarettes (Betts et al., 2021, Colamussi et al., 2007, McClure et al., 2020, Pericot-Valverde et al., 2015, Saladin et al., 2012, Wray et al., 2015, Zanchi et al., 2016), the present study found that exposure to tobacco-related cues elicited a significant and comparable increase in craving among men and women. Extending on previous research, we distinguished between relief and reward craving, potentially offering a more sensitive means of detecting gender differences in cue-induced craving responses (Cox et al., 2001, Dondé et al., 2020). However, based on the current findings, it can be concluded that changes in craving in response to tobacco-related cues, even when distinguishing between relief and reward craving, do not differ between men and women with an TUD, adding to the findings of the majority of studies.

Exposure to tobacco cues significantly reduced HRV, indicating decreased parasympathetic and vagal activity, a marker of heightened stress (Rajendra Acharya et al., 2006). While previous studies in alcohol use disorder populations have shown mixed HRV responses to substance cues (Firdaus et al., 2025, Garland et al., 2012, Ingjaldsson et al., 2003, Rajan et al., 1998), the present findings align with research showing decreased high-frequency HRV and vagal activity following tobacco cue exposure (Kroczek et al., 2024). We demonstrated that HRV decreased during the viewing of smoking-related videos and images, but increased again during the handling of cigarettes, resembling similar effects seen during the handling of alcohol-related paraphernalia in hazardous drinkers (Firdaus et al., 2025). This increase in HRV may reflect a calming effect of handling a cigarette, indicating reward anticipation (Ralevski et al., 2019), or may indicate natural HRV recovery (Mohammadi et al., 2019). Because HRV increases during cigarette handling were significant only in participants with low nicotine withdrawal, the lack of HRV recovery in those with severe withdrawal suggests more maladaptive ANS functioning. These results highlight the importance of measuring HRV across different cue exposure stages to capture dynamic ANS responses. Our findings show that abstinent individuals who smoke cigarettes exhibit clear ANS reactivity to tobacco cues, indicated by HRV reductions, and that RMSSD reliably measures short-term physiological cue reactivity. Notably, nicotine withdrawal symptoms may influence these physiological responses, reinforcing the clinical value of HRV as a biomarker for tobacco cue reactivity. Given its relative cost-effectiveness and greater accessibility compared to fMRI, the current gold standard for measuring (tobacco) cue reactivity, HRV shows strong potential as a practical alternative or complementary biomarker for assessing physiological responses to tobacco cues.

We hypothesized greater exposure-induced HRV decreases in women, reflecting stronger stress responses, and greater HRV increases in men, indicating stronger reward-related responses (Colamussi et al., 2007, McClure et al., 2020, Saladin et al., 2012, Tomko et al., 2020, Wray et al., 2015). However, no gender differences in tobacco cue-induced HRV were found. One explanation is that gender differences in physiological cue reactivity may be more evident in at-risk individuals than those with established SUDs, such as individuals in our sample. Supporting this, neuroimaging studies show stronger cue reactivity in men at risk for alcohol use disorder (Kaag et al., 2019), but not in patients with an alcohol use disorder (Gerhardt et al., 2022). Alternatively, Firdaus et al. (2025) found stronger HRV decreases in non-abstinent hazardous drinking men than women, suggesting gender differences in cue-induced HRV may be more pronounced in non-abstinent substance users compared to the abstinent individuals included in this study. However, since withdrawal symptoms did not differ by gender in our sample and adjusting for them did not change results, they likely do not explain these discrepancies.

While no gender differences were found in overall HRV response, a notable gender-specific pattern emerged in the relationship between subjective craving and physiological cue reactivity. Specifically, in women with TUD, subjective craving was significantly associated with HRV changes, but this relationship was absent in men. This contrasts with earlier neuroimaging findings, where alcohol cue-induced neural activation correlated more strongly with subjective responses in men than women (Seo et al., 2010, Urban et al., 2010). We hypothesized that decreases in HRV would reflect relief craving processes, while increases in HRV would reflect reward craving (Ralevski et al., 2019). However, our results suggest the opposite for women: stronger relief craving was linked to blunted HRV reductions and impaired HRV recovery during cigarette handling, whereas stronger reward craving was associated with greater HRV decreases and more pronounced recovery during handling of tobacco-related paraphernalia. These findings align with a recent study showing blunted HRV reductions in individuals reporting heightened relief craving, though that study did not find gender differences in this association (Firdaus et al., 2025). Since the craving-HRV relationship in women was independent of nicotine withdrawal, the blunted HRV reductions and impaired recovery among women with high relief craving may indicate a persistent stress state that suppresses parasympathetic (vagal) tone, impairing HRV recovery (Kwon et al., 2022). The finding that women reporting greater reward craving exhibited stronger cue-induced HRV reductions and subsequent recovery is more challenging to interpret. Prior research links alcohol cue-induced HRV reductions to compulsive drinking patterns (Ingjaldsson et al., 2003). Moreover, excessive HRV reactivity to emotional challenges may signal disruptions in self-regulatory functioning (Beauchaine and Thayer, 2015). Given that the association between reward craving and HRV reductions was significant in women but not men, these findings suggest that craving intensity in women may reflect a stronger link to dysregulated self-control mechanisms and compulsive substance use behaviors. To conclude, gender differences in cue-induced HRV changes and the associations with cue-induced craving are complex, and their clinical implications remain unclear. However, our findings reveal gender-specific mechanisms in TUD. These findings highlight stronger autonomic nervous system involvement in cue-induced craving in women. This emphasizes the need to consider gender in research and clinical practice, particularly for interventions targeting the ANS, such as HRV biofeedback (Tosti et al., 2024).

To our knowledge, this is the first study showing gender differences in the relationship between subjective tobacco cue reactivity and physiological HRV responses. Some limitations warrant consideration. Although RMSSD is suitable for short-term HRV measurement (Shaffer and Ginsberg, 2017), future research should explore other HRV indexes to identify which best detect gender differences in TUD mechanisms. Moreover, because participants were instructed to abstain from smoking 48 h prior to the first session, we were unable to verify smoking status using biochemical validation, which is a limitation of the current study. Additionally, menstrual cycle phase was not included in the current analyses, despite evidence that fluctuating ovarian hormones substantially impact the mechanisms underlying TUD (Giner et al., 2024), including neural tobacco cue reactivity (Franklin et al., 2022). Moreover, while exploratory analyses on the influence of hormonal contraceptives were performed and described in the Supplementary Materials, the considerable heterogeneity of contraceptive types within the current sample, combined with small subgroup sizes, limits the ability to draw definitive conclusions about their specific effects on tobacco cue reactivity. Taken together, these findings underscore the need for focused research to disentangle how hormonal contraceptive use, menstrual cycle variations, and menopause, influence both subjective and physiological cue reactivity in TUD and SUDs. Moreover, integrating these sex-related biological measures with a composite gender index, reflecting gender-associated variables such as occupation, caregiver roles, and receipt of child support (Cipriani et al., 2024, , … on behalf of the TORSADE Cohort Working Group, 2020), is critical for fully elucidating the complex interplay of sex and gender in addiction-related neurobiological mechanisms. This multidimensional approach will support more nuanced analyses and ultimately inform equitable, evidence-based strategies for prevention and treatment.

To conclude, we showed tobacco cue-induced increases in both reward and relief craving as well as decreases in HRV that were similar for men and for women. However, the association between subjective and physiological tobacco cue reactivity was only significant in women. These findings contribute to a better understanding of gender differences in the mechanisms underlying TUD and underscore the importance of considering gender in both research and clinical practice for TUD.

## Authorship contribution

AK collected the data. LH and AMK performed the analyses and prepared the first draft of the manuscript. AK and TjdV critically reviewed and revised the manuscript.

## CRediT authorship contribution statement

**Hoffman Lucia:** Writing – original draft, Formal analysis, Data curation. **Koomen Annel:** Writing – review & editing, Investigation, Formal analysis, Data curation. **de Vries Taco:** Writing – review & editing, Supervision, Funding acquisition. **Anne Marije Kaag:** Writing – review & editing, Supervision, Funding acquisition, Formal analysis.

## Declaration of Generative AI and AI-assisted technologies in the writing process

Perplexity AI was used under human supervision to rephrase and shorten certain sentences in this manuscript, with the aim of improving clarity and English language quality. All AI-generated suggestions were reviewed and edited by the authors, who take full responsibility for the final content.

## Role of funding source

This work was supported by the Dutch Cancer Society (in Dutch: 10.13039/501100004622KWF Kankerbestrijding; project number 14189) and EMDR Research Foundation.

## Declaration of Competing Interest

All authors declare that they have no conflicts of interest.

## Data Availability

The raw, anonymized, data supporting the conclusions of this article will be made available by the authors, without undue reservation.

## Footnotes

Appendix A Supplementary data associated with this article can be found in the online version at doi:10.1016/j.dadr.2025.100407.
