# Supplementary material for: Exploring gender differences in tobacco cue-induced craving and heart rate variability in individuals with a tobacco use disorder
Source: Drug Alcohol Depend Rep. 2025 Dec 29;18:100407. doi: 10.1016/j.dadr.2025.100407 (PMC12813570; doi:10.1016/j.dadr.2025.100407)
Supplement: Supplementary file 1 — Supplementary material [file mmc1.docx]

# Exploratory analyses on the effect of hormonal contraceptive use on subjective and physiological tobacco cue reactivity

Given evidence that hormonal contraceptives affect craving, withdrawal, stress reactivity, and nicotine metabolism (Allen et al., 2017; Macatee et al., 2024; Stewart et al., 2022), exploratory analyses were performed on the impact of hormonal contraceptive use on tobacco cue reactivity.

Exploratory analyses indicated no differences in tobacco-cue reactivity, subjective or physiological, among men, women using hormonal contraceptives, and naturally cycling women. Moreover, the association between exposure-induced changes in HRV and reward craving did not differ significantly among men, women on hormonal contraceptives, and naturally cycling women (F₂,₆₃=2.93, p=0.061, η²=0.085). However, the relationship between exposure-induced changes in HRV and in relief craving did differ significantly by hormonal status (F₂,₆₃=3.99, p=0.023, η²=0.112). Simple effects indicated no significant association in men (F₃,₃₂=2.73, p=0.108, η²=0.079), but indicated significant, quadratic associations in natural cycling women (F₃,₁₇=6.815, p=0.018, η²=0.286) and linear associations in women on hormonal contraceptives (F₃,₁₄=9.919, p=0.009, η²=0.396).

These exploratory analyses do suggest that (the association between) subjective and physiological tobacco cue reactivity is moderated by the use of hormonal contraceptive use, but does not seem to find blunted cue-reactivity as has previously been demonstrated in a sample of individuals with an cannabis use disorder (Macatee et al., 2024).Given the considerable heterogeneity of contraceptive types within the current sample, combined with small subgroup sizes, limits the ability to draw definitive conclusions about their specific effects on tobacco cue-reactivity.
